# Prevalence and factors associated with symptoms of depression in family members of people hospitalized in the intensive care unit

**DOI:** 10.5935/0103-507X.20220080-en

**Published:** 2022

**Authors:** Luciana Maciel de Souza, Kátia Santana Freitas, Aloísio Machado da Silva Filho, Jules Ramon Brito Teixeira, Geysimara Santos Silveira Souza, Elaine Guedes Fontoura, Alyne Henri Motta Coifman, Pollyana Pereira Portela

**Affiliations:** 1 Postgraduate Program Professional Master’s in Nursing, Universidade Estadual de Feira de Santana - Feira de Santana (BA), Brazil.; 2 Postgraduate Program in Collective Health, Universidade Estadual de Feira de Santana - Feira de Santana (BA), Brazil.; 3 Universidade Federal da Bahia - Salvador (BA), Brazil.

**Keywords:** Depression, Family, Mental disorders, Mental health, Hospitalization, Hospital care, Prevalence, Intensive care units

## Abstract

**Objective:**

To evaluate the prevalence and factors associated with depression in family members of people hospitalized in intensive care units.

**Methods:**

A cross-sectional study was conducted with 980 family members of patients admitted to the intensive care units of a large public hospital in the interior of Bahia. Depression was measured using the Patient Health Questionnaire-8. The multivariate model consisted of the following variables: sex and age of the patient, sex and age of the family member, education level, religion, living with the family member, previous mental illness and anxiety.

**Results:**

Depression had a prevalence of 43.5%. In the multivariate analysis, the model with the best representativeness indicated that factors associated with a higher prevalence of depression were being female (39%), age younger than 40 years (26%) and previous mental illness (38%). A higher education level was associated with a 19% lower prevalence of depression in family members.

**Conclusion:**

The increase in the prevalence of depression was associated with female sex, age younger than 40 years and previous psychological problems. Such elements should be valued in actions aimed at family members of people hospitalized in intensive care.

## INTRODUCTION

When thinking of the family as a group of individuals linked by affective bond and a sense of belonging that may suffer a functional imbalance if a member becomes critically ill, the term Postintensive Care Syndrome-Family (PICS-F) was coined to describe the psychological disorders (anxiety, depression and posttraumatic stress) that affect family members during the patient’s hospitalization and up to 12 months after discharge.^(1-3)^

Among the disorders that make up PICS-F, depression has the greatest disabling potential. Its prevalence can reach 90% and only decrease 5 to 36% six months after discharge.^(4)^ Prevalence rates of 60%^(5)^ and 71.8% are reported in families with members hospitalized in intensive care units (ICUs).^(6)^

Factors associated with PICS-F include discomfort related to hospitalization and feelings related to the patient, personal coping capacity and factors arising from the environment,^(7)^ as well as severity of critical illness, age, sex and clinical conditions, such as the need for mechanical ventilation by the patient and family members’ history of anxiety.^(8^.^9)^

In the first 30 days after ICU discharge, similar symptoms of depression can be identified among patients and their family members. At 90 days, family members had a higher prevalence of depression than the patients. Family members whose patients died had higher levels of depression than family members of survivors.^(10)^ The determinants of the development of depression include factors related to the patient, family and ICU environment and demand a sensitive view of the care team and preventive interventions.

Despite these aspects, family members and patients still experience restrictions on ICU visits due to coronavirus disease 2019 (COVID-19) restrictions. This scenario suggests that social distancing can have a significant impact on family members and patients, a factor that has prompted health institutions to use strategies, such as video calls between family members, patients and care teams, with the objective of improving communication, reducing stress levels and benefitting the mental health of patients and their families.^(11)^

Family-centered care has been prioritized in ICUs due to the importance of family support in patient recovery^(6)^ However, the gap in scientific knowledge related to the national reality hinders the awareness of managers and professionals and the development of preventive strategies. From this perspective, this study aimed to evaluate the prevalence and factors associated with depression in families with members hospitalized in ICUs.

## METHODS

A cross-sectional study that followed the guidelines of the STROBE statement^(12)^ was approved by the Research Ethics Committee (opinion 3,527,238). Data were obtained through structured interviews, which began in 2016 and ended in March 2020, conducted in the adult ICU of a general hospital in a municipality in northeastern Brazil.

To calculate the sample, a finite population of 862 admissions to the ICU per year at the aforementioned hospital was considered; an estimated proportion of 25% of family members with symptoms of depression (based on prevalence assessment studies in the Brazilian context); confidence intervals of 95% (95% CI) and a maximum error rate of 5%, with a total of 218 family members interviewed annually. Considering an additional 10% of losses and refusals, 980 family members of patients admitted to the ICUs were interviewed. Family members who met the following criteria were included: visited the patient in the ICU at least once; were age 18 or older; being one of the closest family members; and having the family member stay in the ICU a minimum of 48 hours. Only one representative per hospitalized person was elected for the study, and family was considered “a group of people linked by affective bond and sense of belonging”.^(1)^

The family members were approached in the ICU waiting room. Those who met the criteria and signed the Free and Informed Consent Form (ICF) participated in the interview and filled out the following: a questionnaire consisting of information from the family member and the hospitalized relative (sociodemographic, clinical and ICU admission data); the Hospital Anxiety and Depression Scale (HADS-A; a subscale of seven items used to measure anxiety, in which a score >10 was considered positive for anxiety);^(13)^ the PHQ-8, used to screen for depression, through eight items that capture the diagnostic criteria of the Diagnostic and Statistical Manual of Mental Disorders-5th edition (DSM-5), in which a score ≥ 10 points was considered positive for depression.^(14,15)^

**Figure 1:**
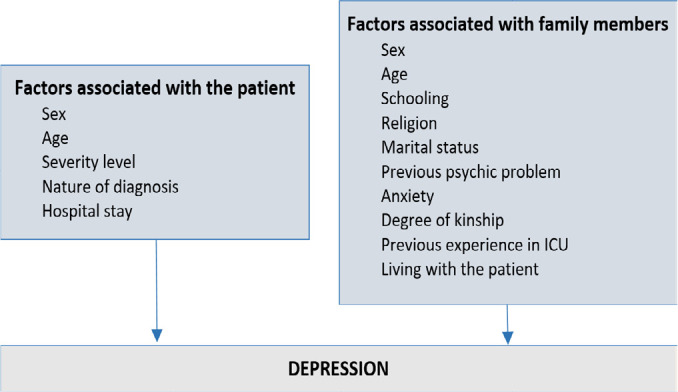
Conceptual model for the analysis of factors associated with depression in family members in the intensive care unit

The absence of records of severity indices in the unit led to the classification of the patient’s severity level between low (stable or hemodynamically compensated) and high (hemodynamically unstable).^(16)^ The variables investigated were selected from the survey in the scientific literature and schematized according to the theoretical model shown in figure 1.

ICU - intensive care unit.

Data collection and typing were performed by a team of scholars from the nursing, medicine and psychology courses, who were trained to standardize the procedures for collecting, storing and protecting information. The data were retained in the Statistical Package for the Social Sciences (SPSS), version 22.0. Descriptive statistics were used to analyze the characteristics of the population using absolute and relative frequencies. For bivariate analysis, the variables were dichotomized, and the prevalence ratios and the respective 95%CIs were estimated.

In the multivariate analysis, confirmatory regression analysis was used to identify potentially effect-modifying and confounding variables. For the testing of modifying variables, the Breslow‒Day test of homogeneity was performed, with a p value ≤ 0.05, and those that showed evidence of statistical interaction in the stratified and multivariate analysis were considered. The Mantel‒Haenszel method was used to test confounders, which considers the variation (∆%) between the crude and adjusted prevalence ratios with a significance ≥10%.

After defining the confounders and effect modification and the subsequent separation of these variables, multivariate regression analysis (Poisson with robust variance) was performed to determine the final models. Variables with p values ≤ 0.20 in the bivariate analysis were included in this model. After defining the complete model, the variables with the highest nonsignificant p value were excluded until the lowest value of the Akaike information criterion (AIC) was obtained. The AIC variation was used to define the exclusion (decrease in value) or maintenance (increase in value) of each variable in the model. In the latter case, the variable was considered a confounder, which was maintained in the model for adjustment. This procedure was repeated until the variables with p ≤ 0.05 remained in the model.

At the end of this stage, the final model of factors associated with depression among family members was constructed (Model A). Subsequently, two models stratified by sex were evaluated to identify possible differences in associated factors between men (Model B) and women (Model C). A statistical significance level of 5% was adopted. For the multivariate analyses, Stata software version 14 was used.

## RESULTS

A total of 980 family members participated in the study. The hospitalized individuals had a mean age of 50.3 years (standard deviation - SD of 20.0), were predominantly male (59.7%), had stable severe disease (47.4%), had a clinical diagnosis (52.8%) and had a mean hospital stay of 5.7 days (SD 7.2). The family members had a mean age of 40.4 (SD 13.0), were female (77%), had completed high school (48.4%), were married (41.7%), were Catholic (47.8%), were economically active (37.6%), did not live with the hospitalized family member (54.6%) and had children (34.4%) or siblings (19.8%). A total of 93.5% had no previous mental health problems, and 68.3% reported no experience of hospitalization of other family members in the ICU and had made approximately 4.6 visits (SD of 3.1) by the time of the interview. Depressive symptoms were detected in 43.5% of the family members.

Depression was more prevalent among family members of male, elderly patients, with a higher level of severity and surgical diagnosis (Table 1).

**Table 1:** Prevalence of depression according to sociodemographic and clinical characteristics of people hospitalized in the intensive care unit

Variables	n (%)	Prevalence	p value^*^	PR	95%CI
Sex (n = 980)			0.160		0.92 - 1.55
Male (585)	265 (27.04)	45.3		1.20	
Female (395)	161 (16.42)	40.1			
Severity level (n = 945)			0.351		0.86 - 1.50
High (640)	285 (30.15)	44.53		1.14	
Low (305)	126 (13.33)	41.31			
Nature of diagnosis (n = 977)			0.417		0.69 - 1.16
Clinical and clinical that progressed to surgery (582)	247 (25.28)	42.44		0.89	
Surgical (395)	178 (18.21)	45.06			
Age (n = 979)			0.001		1.12 - 1.55
Until 59 years (645)	305 (31.15)	47.29		1.31	
From 60 years (334)	120 (12.25)	35.93			
Length of stay (n = 971)			0.546		0.84 - 1.39
Over 5 days (469)	208 (21.42)	44.35		10.8	
Up to 4 days (502)	213 (21.93)	42.43			

PR - prevalence ratio; 95%CI - 95% confidence interval.

* Pearson’s chi-square test of independence, considering p < 0.20 for entry into the multivariate model.

Depression was predominant among female family members, with lower education levels, married or in a stable relationship, without religion, economically inactive, living with the hospitalized relative, and with previous mental health problems and anxiety symptoms (Table 2).

**Table 2:** Prevalence of depression according to sociodemographic and clinical characteristics of family members in the intensive care unit

Variables	n (%)	Prevalence	p value^*^	PR	95%CI
Sex (n = 980)			0.000		1.52 - 2.44
Female (755)	369 (37.65)	48.87		1.92	
Male (225)	57 (5.8)	25.33			
Age (years) (n = 979)			0.108		0.77 - 1.02
Less than 39 (494)	202 (20.63)	40.89		0.88	
From 40 (485)	223 (22.77)	45.98			
Schooling (n = 979)			0.003		0.69 - 0.92
Average or higher (654)	263 (26.86)	40.21		0.18	
Up to elementary (325)	163 (16.64)	50.15			
Marital status (n = 980)			0.507		0.71 - 1.18
Married or stable union (573)	244 (24.89)	42.58		0.91	
Single or divorced (407)	182 (18.57)	44.72			
Religion (n = 980)			0.080		0.98 - 1.41
No (150)	75 (7.65)	50.00		1.18	
Evangelical, Catholic and others (830)	351 (35.81)	42.29			
Work situation (n = 979)			0.329		0.88-1.46
Unemployed, retired or housewife (440)	199 (20.32)	45.23		1.13	
Active, self-employed and others (539)	227 (23.18)	42.12			
Reside with the patient (n = 973)			0.005		1.06 - 1.41
Yes (438)	211 (21.68)	48.17		1.22	
No (535)	210 (21.58)	39.25			
Degree of kinship (n = 979)			0.497		0.84 - 1.40
Spouse or child (520)	231 (23.59)	44.42		10.9	
Father, mother or brother (459)	194 (19.81)	42.27			
Previous mental disorder (n = 979)			0.000		1.57 - 2.16
Yes (63)	48 (4.9)	76.19		1.84	
No (916)	378 (38.61)	41.27			
Experience of other relatives in the ICU (n = 971)			0.794		0.78 - 1.36
Yes (302)	130 (13.38)	43.05		1.03	
No (669)	294 (30.27)	43.67			
Anxiety (n = 980)			0.000		3.94 - 5.26
Yes (481)	343 (35.0)	71.31		4.28	
No (499)	83 (8.46)	16.63			

PR - prevalence ratio; 95%CI - 95% confidence interval; ICU - intensive care unit.

* Pearson’s chi-square test of independence.

The multivariate model comprised the variables patient sex, patient age, family member sex, family member age, education level, religion, living with the family member, previous mental illness, and anxiety. In the Breslow‒Day homogeneity test, no variable was identified as an effect modifier. In the confounding analysis, the variable anxiety was identified as a confounder (∆ = 27.6%); therefore, it was included in the multiple analysis only to fit all models. Table 3 shows the multivariate model for factors associated with depression in the study population.

**Table 3:** Multivariate models of factors associated with depression in family members of people hospitalized in the intensive care unit

Variables	Model A^*^		Model B^*^†		Model C‡§	
	PR	95%CI	PR	95%CI	PR	95%CI
Education						
Until fundamental	1.00	-	-	-	1.00	-
Medium or higher	0.81	0.72 - 0.91	-	-	0.80	0.71 - 0.90
Age of the family member (years)						
From 40	1.00	-	1.00	-	1.00	-
Below 39	1.26	1.09 - 1.44	1.64	1.02 - 2.62	1.23	1.07 - 1.41
Previous psychological problem						
No	1.00		1.00		1.00	
Yes	1.38	1.20 - 1.58	1.65	1.17 - 2.30	1.34	1.17 - 1.54
Sex of the family member						
Male	1.00	-	-	-	-	-
Female	1.39	1.13 - 1.73	-	-	-	-
	AIC = 1.398		AIC = 1.056		AIC = 1.506	

PR - prevalence ratio; 95%CI - 95% confidence interval; AIC - Akaike information criterion.

* Model adjusted for anxiety and living with the hospitalized person; † model adjusted for male sex; ‡ model adjusted for anxiety; § model adjusted for female sex.

In the multivariate analysis of Model A, the variable residing with the hospitalized person was added to the adjustment due to the increase in the AIC after its withdrawal. In this model, family members with higher education had a prevalence of depression 19% lower than those who had up to an elementary school level of education (prevalence ratio, a PR of 0.81; 95%CI 0.72 - 0.91). Family members aged up to 39 years had a 26% higher prevalence of depression than family members aged 40 years and older (PR 1.26; 95%CI 1.09 - 1.44). Having a previous mental health problem resulted in a 38% increase (PR 1.38; 95%CI 1.20 - 1.58); females increased 39% (PR 1.39; 95%CI 1.13 - 1.73) on the prevalence of depression. Based on these results, we chose to stratify the analysis by sex to assess the associated factors from a gender perspective.

The model stratified by male sex (Model B) was adjusted for the variables anxiety and living with the hospitalized person. The variable education level was excluded from the model using the criterion of increasing the AIC. Family members up to 39 years of age had a 64% higher prevalence of depression than family members aged 40 years and older (PR 1.64; 95%CI 1.02 - 2.62); in addition, having a previous mental illness resulted in a 65% increase (PR 1.65; 95%CI 1.17 - 2.30) in the prevalence of depressive symptoms in men.

The model stratified by female sex (Model C) was adjusted for the variable anxiety. The variable residing with the hospitalized person was excluded by the criterion of increased AIC. Female family members with higher education (high school or college) had a 20% lower prevalence of depression than those with less education (PR 0.80; 95%CI 0.71 - 0.90). Younger family members had a 23% increase in the prevalence of depression (PR 1.23; 95%CI 1.07 - 1.41). Having a previous mental disorder increased its prevalence in women by 34% (PR 1.34; 95%CI 1.17 - 1.54).

As Model A was not stratified, it was considered the final model to justify the factors associated with depression in family members of people hospitalized in the ICU in the sample evaluated.

## DISCUSSION

This study showed the psychological burden of family members during the hospitalization of a relative in the ICU. The overall prevalence of depression in the sample evaluated was 43.5%, a high rate compared to the 5.8% prevalence of depression in the general Brazilian population in 2015.^(17)^ The factors associated with a higher prevalence of depression were age up to 39 years, female sex and previous mental illness. Higher education was associated with a lower prevalence of depression.

In international contexts, a high prevalence of depression was identified in families in the context of the ICU. In U.S. studies, rates ranged from 10.3%, ^(18)^ 14%,^(19)^ 16%^(20)^ to 20%;^(21)^ while in Greece, the percentage was 49.1%^(5)^ and in Turkey, 71.8%.^(6)^ The lower international prevalence of depression can be explained by the high levels of satisfaction with the care provided, with the competence of the team^(18)^ and with the emotional support provided to family members, which allows for better decision-making and coping with adverse situations resulting from critical illness.^(19)^ The higher prevalence rates can be attributed to institutional characteristics and regional differences.^(5)^

In Brazil, most of the prevalence rates identified were below the values found in this study, ranging from 6.5% to 28.9%.^(10,22-25)^ This disparity can be understood by the adoption of a policy encouraging the presence of the family in the ICU, such as 24-hour visits,^(22)^ in addition to other family support measures.

The highest national prevalence was 54.3%, found in a study conducted in the ICU of a public hospital in São Paulo, which can be explained by factors related to 1) the environment (level of severity, high rates of sepsis and mortality in the sector, inadequate physical structure, such as the lack of a waiting room and curtains to separate beds); and 2) family members (low education levels resulted in greater difficulty in understanding the diagnosis and prognosis).^(25)^ The high prevalence of depression in these family members reflects the lack of care and a less sensitive approach to the specific needs of this group.

The factors associated with a higher prevalence of depression were age up to 39 years, female sex and previous mental illness. Higher education was associated with a lower prevalence of depression.

These results corroborate recent studies that reinforce female sex as an exposure factor for developing symptoms of depression.^(22)^ Depressive disorders in different sexes have been investigated in several studies, which indicate a prevalence in women that can reach twice that of men. The causes of this disparity can be attributed to several factors, such as hormonal factors, which explains the higher incidence among women after puberty, and the balance between the two sexes when women reach menopause.^(26)^ The inflammatory response resulting from stressful stimuli is associated with the development of depressive symptoms. Although they produce proinflammatory cytokines in similar amounts as men, women are more sensitive to stress situations associated with the emergence of depressive mood and social distancing.^(27)^

Issues related to social gender roles may also be associated with the high prevalence of depression among women, according to a study that found a prevalence of 26.8% among males and 40.4% among females. In this sample, participants who reported greater dissatisfaction with unequal sex roles in politics and in family roles had higher rates of depression.^(28)^

The relationship with education level was opposite to the findings in the scientific literature, whose results indicate a greater number of symptoms of depression related to a higher level of education in a sample of family members in the ICU of a large private hospital in São Paulo (SP).^(29)^ In the general population, a study conducted in Germany showed a relationship between a high level of education and the development of depressive symptoms in a 2-year and 6-month follow-up period in a sample that did not present symptoms at the initial evaluation.^(30)^

The association between a lower prevalence of depression and a higher level of education can be understood by the greater understanding of the information transmitted by the health team, which reduces the anguish and uncertainty regarding the relative’s health status; the ease of access to information about diagnosis and treatment; and by the greater search for support networks to encourage resilience in this scenario.

Studies on the association between previous mental illness or the age of the family member and the prevalence of depression in the ICU were not identified. However, a recent study found an association between younger individuals and psychological stress during the COVID-19 pandemic,^(31,32)^ which can be explained by the greater capacity for resilience acquired throughout life by younger individuals. Additionally, in the general population in the context of a pandemic, people with a previous psychiatric history were more vulnerable to developing symptoms of depression.^(32)^

The analysis stratified by sex made it possible to identify the factors associated with each group. For males, the level of education showed no association with the development of depression. However, the age of the family member and previous mental illness were associated with this outcome and represented increases of 64% and 65%, respectively. For females, younger family members had a 23% increase in the prevalence of depression and with the presence of previous mental illness, 34%. The highest level of education for this group acted as a protective factor, with a 20% reduction in prevalence compared to individuals with less education.

These results highlight the need for effective support for the family in this stressful and challenging scenario. The factors associated with depression can be considered intrinsic because they are not directly related to any organizational aspect of the unit itself. However, this does not exempt the institution from the responsibility of planning and implementing measures to prevent this problem from affecting the mental health of family members.^(33)^ When including the family in the care plan, it should be considered that each family has specificities, with its own functioning, and each member has unique manifestations of psychological distress; considering these individual needs can make all the difference.

The relevance of the findings of this study in the post-COVID-19 pandemic scenario, which led to a restructuring of the way of life around the world, is highlighted. The rapid spread of the virus and the great potential for systemic complications challenged health policies and continues to require a restructuring of teams and institutions, which need to rethink ways of dealing with the family. Severe restrictive measures are part of the new ICU safety protocols and can lead to exhaustion of the entire family nucleus; therefore, they require incisive measures to prevent the development of depression.^(34)^

It is up to hospital management to seek the most appropriate strategies to promote better coping with the illness process by the family, based on the perception of health professionals about family members’ needs for support. Interventions have shown satisfactory results in the reduction of stress levels, with improvement of mental health as a result of strategies such as talk groups directed by psychology and nursing professionals, in which family members share their experiences, needs and resources.^(34)^

Allowing family members to meet the team responsible for the care of their relative and to feel free to ask questions has been an effective strategy to increase satisfaction. Therefore, a moment of dialog should be reserved with professionals from each category involved in patient care. Occupational therapy can encourage the use of recreational resources, such as art therapy, music therapy and encouragement of spirituality, to promote greater resilience.^(35)^

The main limitation of this study was that the study was unicentric, which compromises the generalization of the findings to different realities. The evaluation of the interviewees in a single moment, without subsequent follow-up, limited the understanding of the development patterns and duration of the disease.

## CONCLUSION

Female sex, previous mental illness and age younger than 40 years were associated with an increased prevalence of depression, while higher education was associated with a lower prevalence. Regarding gender differences, depression in men and women was associated with younger age and previous mental health problems. However, among them, higher education level acted as a protective factor for depression.

This topic opens space for new research, especially focused on the mental health of family members living in public and private intensive care units and, above all, considering the hospitalization of people with COVID-19. The recent emergence of this disease has had a strong impact on mental health worldwide and accentuated the gap in the literature, reinforcing the need for intensive care professionals to be attentive to ensure the inclusion of family members in their treatment plans.
